# Phase Equilibrium and Interdiffusion in Blends of Polystyrene with Polyacrylates

**DOI:** 10.3390/polym13142283

**Published:** 2021-07-12

**Authors:** Uliana V. Nikulova, Anatoly E. Chalykh

**Affiliations:** Frumkin Institute of Physical Chemistry and Electrochemistry Russian Academy of Sciences (IPCE RAS), 31, bld.4 Leninsky prospect, 119071 Moscow, Russia; chalykh@mail.ru

**Keywords:** polystyrene, polyacrylate, polymethyl acrylate, polyethyl acrylate, polybutyl acrylate, polyethylhexyl acrylate, phase state diagram, diffusion

## Abstract

The solubility and interdiffusion of polystyrene (PS) with polymethyl acrylate (PMA), polyethyl acrylate (PEA), polybutyl acrylate (PBA), and polyethylhexyl acrylate (PEHA) have been studied by the optical interferometry method. Phase state diagrams are plotted. It is shown that they are characterized by the upper critical solution temperatures (UCST), which are localized in the temperature range above 450 K. Pair interaction parameters and their temperature dependences are determined and analyzed. Extrapolation of the temperature dependence of the interaction parameter was used to construct the dome of binodal curves and determine the spinodal curves in the framework of the Flory–Huggins theory. The diffusion coefficients of polystyrene into polyacrylates and polyacrylates into polystyrene are calculated. The dependences of the interdiffusion coefficients on the concentration, temperature, polystyrene molecular weight, and the number of carbons in the side chain of polyacrylate are analyzed. The numerical values of the interdiffusion coefficients of PS-1 into polyacrylates at 433 K change as −8.5 → −6.7 → −6.4 in the homologous series PMA → PEA → PBA. The coefficients of friction are calculated and the effect of change in the matrix structure on the diffusion of polystyrene in them is estimated.

## 1. Introduction

Among the numerous binary mixtures of polymers, the Polystyrene (PS)–Polyacrylates (PA) systems have been the subject of constant research for more than 30 years from the standpoint of the thermodynamics of components mixing, technological compatibility, peculiarities of their colloidal-chemical and adhesive properties, phase structure, and the structure of transition interphase layers [1,2,3,4,5,6,7,8,9,10,11,12,13,14,15,16,17,18]. Nevertheless, information on the phase state diagrams of these systems, which is so necessary for the selection and substantiation of the composition and optimal supramolecular organization of this class of composite materials, adhesives, elements of transdermal systems, is fragmentary, contradictory, and insufficient.

So, until recently, PS–PMA, PS–PEA, PS–PBA, PS–polymethyl methacrylate (PMMA), and PS–polybutyl methacrylate (PBMA) mixtures were considered “completely incompatible” [13,14]. This conclusion was made on the basis of determining the solubility of melts and solutions of polymer blends with a molecular weight (MW) of more than 10^5^. In the work [15], by the method of “titration of a PBA melt with MW = 1.19 × 10^5^″, the limit of its compatibility with PS MW = 5.2 × 10^5^ was determined at the level of ~0.001 mass. % at a temperature of 423 K. In the classical works of Kuleznev [16,17], the influence of the MW of components on the limit of their solubility of PS with MW from 2.6 × 10^3^ to 5.5 × 10^5^ in PMMA with MW 8.7 × 10^4^ is described. For the first time at that time, the authors discovered a noticeable increase in the mutual solubility of polymers in the region of low molecular weights characteristic of oligomers.

In [18], fragments of the phase state diagrams of binary PS–PMMA and ternary systems PS–PMMA–Styrene, PS–PMMA–block copolymer of styrene and methyl methacrylate are presented. It was shown that all the listed systems in a wide range of temperatures and compositions are characterized by diagrams of amorphous separation with the upper critical solution temperature (UCST), and the position of the binodal curves in the temperature-concentration field of the diagrams is determined mainly by the ratio of the molecular weight of the components. In [19,20,21,22], we generalized data on mixing PBA and PS, plotted fragments of binodal curves, calculated pair interaction parameters, determined partial translational diffusion coefficients, and activation energies for diffusion of macromolecules migrating in different media.

An important direction in polymer materials science is associated with the copolymerization of PA with polymers such as PS, PMMA, polyacrylic acid (PAA), polyvinyl acetate (PVA), the formation of stable nanosized latexes [1,2,3,5,7,8,11], mixed solutions systems for the manufacture of fiber mats by electrospinning [9], and drug carriers [23]. The authors of these studies also proceeded from the assumption of the incompatibility of polymers. In [17], it was proposed that an equimolecular copolymer of methyl methacrylate (MMA) and butyl acrylate (BA) be considered as “a mixture of many chemically homogeneous copolymers”. At the same time, the authors note that copolymers are homogeneous only in a narrow range of compositions less than 25 mass % PS, and with a higher PS content in copolymer samples, microphases with different degrees of dispersion and different compositions arose.

In our works [19,20,21,22], we also evaluated the effect of the macromolecules’ architecture and the composition of acrylic copolymers on solubility and interdiffusion when mixed with PS. The obtained data allowed us to assert that the macromolecules’ architecture of the copolymer has a smaller effect on the thermodynamic compatibility of polymers as compared to the molecular weight of the diffusant.

Separately, [21] should be noted, which is devoted to the analysis of the phase structure of the block, gradient and statistical copolymers of *n*-butyl acrylate and styrene of different composition and the architecture of macromolecular chains. It was shown for the first time that their block and gradient copolymers are characterized by the presence of a nanosized domain structure with domain sizes from 2 to 20 nm, the length of interphase boundaries on the order of 3–4 nm, and the distribution density of segments over the cross section of a dispersed microphase was also estimated. Such block copolymers with a styrene content of more than 0.4 mole fraction have two glass transition temperatures corresponding to different compositions of coexisting phases, and the gradient copolymer is characterized by the same glass transition temperature, loose packing of domains and sizes of interphase boundaries 4–5 nm. It was proposed to consider the gradient copolymer as a symbiosis of block and statistical copolymers, and to attribute the statistical piece of the gradient copolymer macromolecules to the composition of the interphase. In this case, the density distribution at the edge of the domain is described by an equation similar to the equation for the density distribution of segments in a macromolecular coil.

Despite the fact that the statistical copolymers PBA and PS were among the first on which the method of labeled atoms was tested for the experimental evaluation of the self-diffusion coefficients of macromolecules [24], their translational mobility was practically not studied in the future. Only in works [4,6,10], with the use of NMR in a pulsed gradient and by the method of laser microinterferometry [20,22], was a comprehensive study of the diffusion and solubility of PSs of various molecular weights in block and gradient copolymers of *n*-butyl acrylate carried out, self-diffusion coefficients of PBA colloidal particles measured, the partial diffusion coefficients of PS macromolecules into the matrix of the copolymer of PBA and PS and of the copolymer into the matrix of PS determined, the coefficients of friction of macromolecules and monomer units calculated, and the activation energies of the translational mobility of macromolecules determined.

In the development of these studies, it seemed interesting to expand the measurement range and conduct a detailed study of phase equilibria and interdiffusion in PS–PA systems with different values of side radicals in the chain in a wide concentration, temperature, and molecular weight, which was the purpose of this work.

## 2. Experimental

Polystyrene (PS) and such polyacrylates as polymethyl acrylate (PMA), polyethyl acrylate (PEA), poly-*n*-butyl acrylate (PBA), and polyethylhexyl acrylate (PEHA) were used as objects of research. Such characteristics as the average molecular weight M_w_ (g/mol), polydispersity index M_w_/M_n_ (the ratio of the weight average to the number average), glass transition temperature T_g_, the amount of carbon in the side chain of the polyacrylate, the molecular weight of the polymer’s monomer unit, and polymer density ρ are presented in Table 1.

### 2.1. Sample Preparation

All measurements were carried out on samples with a thickness of 100–120 μm.

### 2.2. Characterization Method

The solubility of the components was determined by *optical interferometry* [26]. The method is based on the principle of in situ registration of optical density distribution in the area of polymer coupling in the process of interdiffusion. The measurement method consisted of fixing a PA sample of 3 × 10 mm^2^ between the diffusion cell glasses, the inner surfaces of which are covered with a layer of translucent metal (Ni + Cr) with a high reflection index. Special fixtures were used to put the sample into optical contact with the surface of the plates, while a small wedge angle θ ≤ 5° was established between the glasses. The polymer sample was placed in a flat capillary of the diffusion cell so that the interference strips oriented parallel to the edge of the wedge were perpendicular to the diffusion front. After assembly, the cell was thermoregulated at a set temperature for at least 30 min. Then, at a lower temperature corresponding to the temperature of the experiment, the PS melt was drawn into the rest of the space by capillary forces. The moment of contact of the components in the diffusion cell was observed in the field of a microscope and recorded as the beginning of the interdiffusion process.

All measurements were carried out on an ODA-2 IPCE diffusiometer (IPCE RAS, Moscow, Russia) [27] in the temperature range from 300 to 440 K. A helium-neon laser (λ = 632.8 nm) was used as a light source. Methods of processing of the interferograms, interdiffusion zones, and phase diagrams construction did not differ from those described earlier [27,28,29].

**Figure 1:**
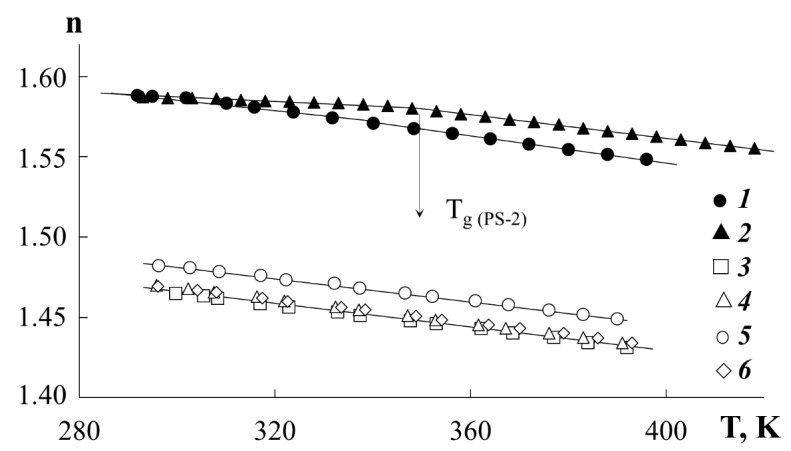
Temperature dependences of the refractive index for PS-1 (1), PS-2 (2), PBA (3), PEA (4), PMA (5) and PEHA (6).

*Refractometry*. Information about the temperature dependences of the refractive indices of polymers is of fundamental importance in interpreting the optical density distribution profiles in the interdiffusion zone. The temperature dependences of the refractive index for polyacrylates and polystyrenes were determined by preliminary experiments (Figure 1). The measurements were carried out on an ATAGO NAR-2T refractometer (ATAGO CO., LTD., Tokyo, Japan) in the temperature range from 290 to 430 K. For all investigated components, linear temperature dependences of the refractive index were observed. In this case, the numerical values of the refractive index for PEA, PBA, and PEHA are close to each other, while for PMA they are somewhat higher. Curves 1 and 2 (Figure 1) clearly show a characteristic break in the curves corresponding to the glass transition temperature of the PS. In the range 370 to 430 K, a large difference is observed in the values of the PS-1 and PBA refractive indices for example (Δn is about 0.119). An increment of the refractive index per interference strip equal to ~ 0.025 provides a sufficiently high sensitivity in identifying changes in the composition in the phase conjugation zone. This information is of fundamental importance in calculating the compositions of coexisting phases in systems with amorphous separation.

The thermal stability of homopolymers was determined by *thermogravimetry* (Netzsch TG209F1 Iris, Germany) at a linear heating rate of 10 K/min. The results of the onset of thermal destruction processes were used to plot the corresponding zones on the phase state diagrams.

## 3. Results and Discussion

### 3.1. Conjugate Phase Zones

**Figure 2:**
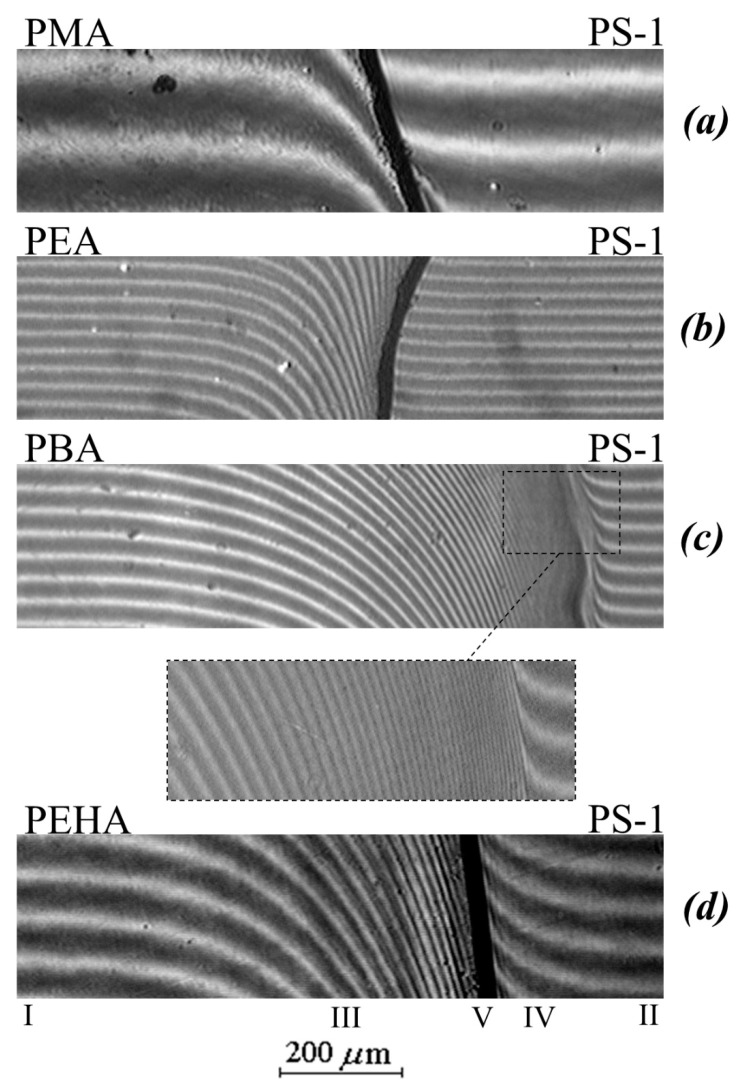
Interferograms of the interaction of PS-1 with PMA (**a**), PEA (**b**), PBA (**c**), and PEHA (**d**) at 433 K.

**Figure 3:**
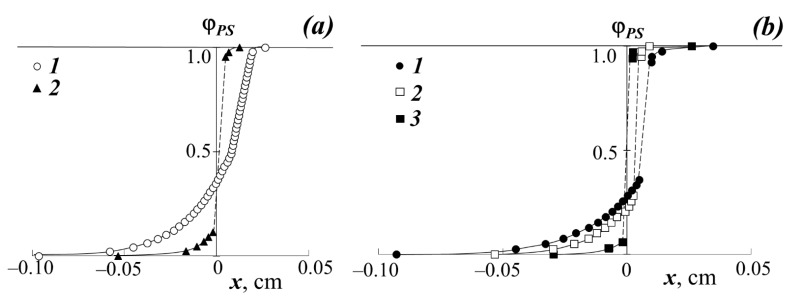
Concentration profiles at 433 K: (**a**) when mixing PBA with PS-1 (1) and PS-2 (2); (**b**) when mixing PS-1 with PEHA (1), PEA (2), and PMA (3).

Figure 2 shows typical interferograms of the interdiffusion zones that spontaneously arise upon conjugation of the PA phases (left) with the PS phase (right) at different temperatures and the molecular weight of the homopolymer. Figure 3 shows the concentration profiles corresponding to the interferograms.

It can be seen that in the case of a low molecular weight of PS (M_w_ < 1.2 × 10^3^) at temperatures above 443 K, the interdiffusion zone is a classic example of completely mutually soluble homopolymers (Figure 2c), which is expressed in a continuous change in the refractive index of gradient systems upon going from one component to another. In this PS–PBA system, in the interdiffusion zone, the formation of a continuous concentration distribution profile is observed going from PS to PBA. Both branches of the concentration profile are symmetric with respect to the midpoint of the ordinate (Figure 3a).

An increase in the molecular weight of PS to 4 × 10^3^ and a transition to other polyacrylates (PMA, PEA, PEHA) change the structure of the transition region of phases conjugation (Figure 2a,b,d). In the middle region of the compositions of the PS–PMA, PS–PEA, PS–PBA, PS–PEHA systems, there is an interphase boundary separating the region of dissolution (diffusion region) of PS macromolecules in the polyacrylate phase (to the left of the phase boundary) from the region of dissolution of polyacrylate macromolecules in the polystyrene phase (to the right of the phase boundary), which indicates the limited solubility of the components. For example, the interdiffusion zone for samples with PS-1 at temperatures from 363 to 433 K is a superposition of three regions: the regions of pure PEHA and PS components (I and II, respectively), the interphase boundary (V), and the regions of PEHA dissolution in PS (IV) and PS in PEHA (III). A similar situation is observed in the PMA–PS, PEA–PS, PBA–PS-2 systems. It is interesting to note that the solubility of PA in PS is characteristic only for the PEHA–PS-1 system (Figure 2d) while the diffusion of PMA and PEA in PS is extremely weak (Figure 2a,b). Such systems are usually referred to as systems with one-sided diffusion. [28,30]. Similar interference pictures and distribution profiles of the composition were observed at lower temperatures of the interdiffusion process.

Under isothermal conditions, near the interphase on both sides of it, the concentrations of φ′ and φ″ corresponding to the compositions of the coexisting phases are established and remain unchanged throughout the observation process, and these are limiting solubility at a given temperature of PS in PA and PA in PS. The compositions of the coexisting phases that are established at the interphase were determined by the method of processing concentration profiles described in [19,26,28]. This structure of the transitional concentration-gradient zone is common and is observed for all studied pairs of PS–PA. The specificity of a particular system is manifested in the length of the interdiffusion regions and the numerical values of the compositions of the coexisting phases.

It was found that the phase boundary in the interferograms under isothermal conditions does not change its position over time, and the concentration jump at the interphase remains constant and depends only on temperature. In the cycles of increasing–decreasing temperature, the jump in the concentrations φ′ and φ″ is quantitatively reproduced, which allows us to speak of equilibrium, which is established at the interphase between the compositions of the coexisting phases.

**Figure 4:**
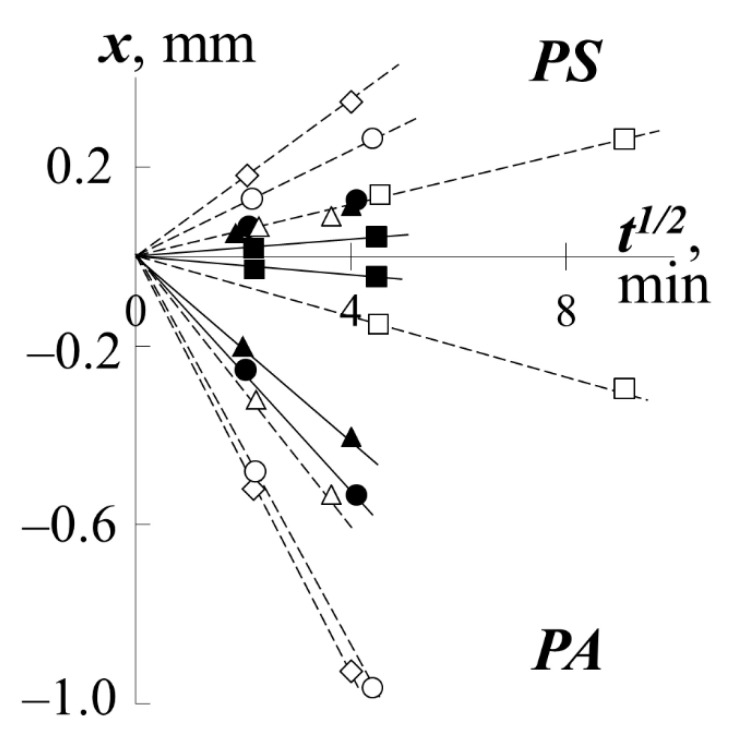
Kinetics of diffusion fronts motion for systems with PS-1 (white dots) or with PS-2 (black dots) when they mixed with PMA (squares), PEA (triangles), PBA (circles), PEHA (rhombuses) at 433 K.

In contrast to the interphase (V in Figure 2d), for the regions of spontaneous mixing of components III and IV, a continuous change in time was recorded in both their sizes and the concentration distribution profile. In this case, each point of the concentration profiles changes its position on the diffusion coordinate (X) strictly in accordance with the diffusion law X–t^1/2^ (Figure 4), i.e., the diffusion mechanism of polymer mixing is observed.

It is interesting to note that the Matano–Boltzmann plane does not change its position in the process of diffusion mixing of homopolymers. This demonstrates an important experimental fact—the invariability of the melt volume upon mixing the polymers.

### 3.2. Phase State Diagram

**Figure 5:**
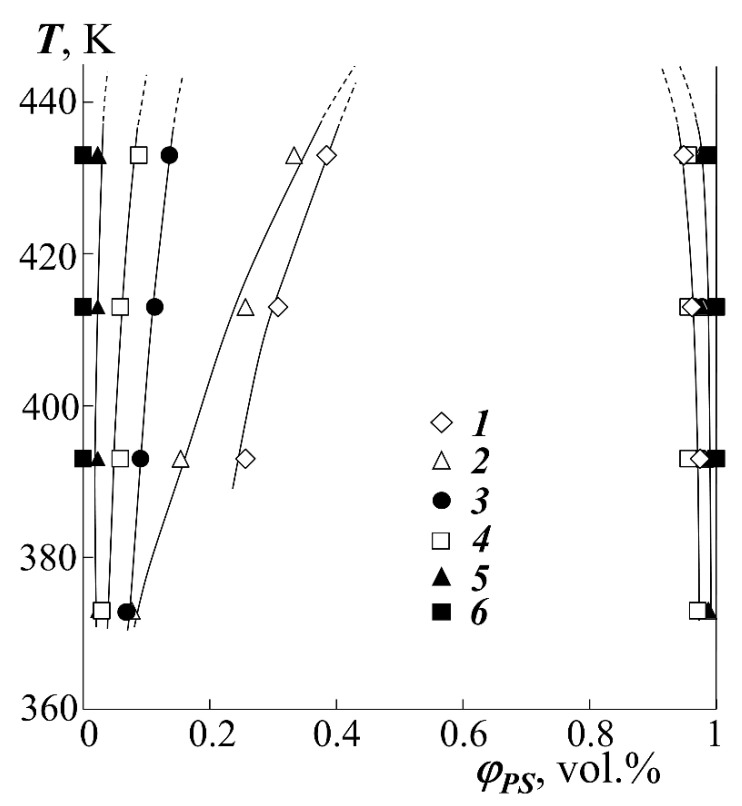
Fragments of binodal curves for the systems PEHA–PS-1 (1), PEA–PS-1 (2), PBA–PS-2 (3), PMA–PS-1 (4), PEA–PS-2 (5) and PMA–PS-2 (6).

Based on the compositions of the coexisting phases φ′(T) and φ″(T) for all studied systems, fragments of binodal curves were constructed (Figure 5). From the formal point of view, all studied systems are characterized by an increase in the mutual solubility of homopolymers with increasing temperature, which indicates the upper critical solution temperature of the components (UCST). However, it was not possible to experimentally determine the position of the critical temperature for most systems, since for them the UCST is higher than the temperature of thermal destruction (T_d_).

In order to construct the dome of binodal curves, we carried out a thermodynamic analysis of experimental data in accordance with the previously developed and tested method for interpreting the phase state diagrams of amorphous separation [18,29].

**Figure 6:**
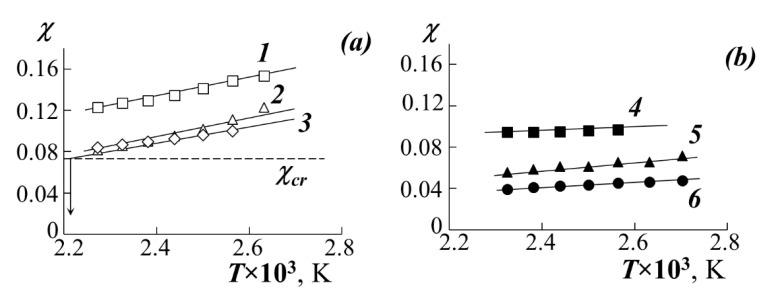
Temperature dependences of *χ* when mixing PMA (1), PEA (2), PEHA (3) with PS-1 (**a**) and, PMA (4), PEA (5), PBA (6) with PS-2 (**b**).

**Figure 7:**
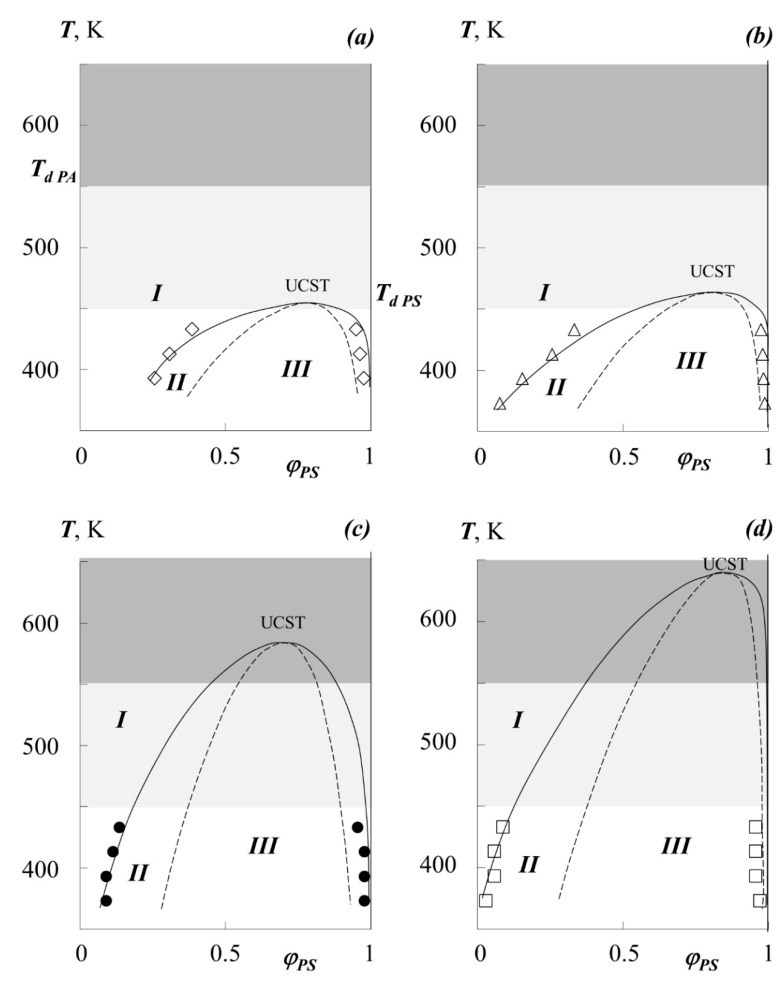
Simulation of phase state diagrams in the framework of the Flory–Huggins theory (see text for details) for the systems PEHA–PS-1 (**a**), PEA–PS-1 (**b**), PBA–PS-2 (**c**), PMA–PS-1 (**d**). Dots are experimental data, solid lines present binodal curves, dashed lines are spinodal curves, light gray area is the zone of thermal destruction of oligomeric polystyrene, dark gray area is the zone of thermal destruction of polyacrylates, I–region of homogeneous state, II–metastable region, III–labile structures region.

- At the first stage of the analysis of the diagrams by Equation (1) and the coexisting phase compositions, the values of the Flory–Huggins parameter χ were estimated under the assumption of the absence of its concentration dependence.
  (1) $$\chi =\frac{\frac{ln\left(\varphi_{1}^{\prime \prime }/\varphi_{1}^{\prime }\right)}{r_{1}}-\frac{ln\left(\varphi_{2}^{\prime \prime }/\varphi_{2}^{\prime }\right)}{r_{2}}}{2\left(\varphi_{2}^{\prime }-\varphi_{2}^{\prime \prime }\right)}$$
  The obtained values of *χ* are the averaged value for the paired parameters of the interaction $\chi_{12}=\chi +\frac{\partial \chi }{\partial \varphi_{1}}\varphi_{1}$ and $\chi_{21}=\chi +\frac{\partial \chi }{\partial \varphi_{2}}\varphi_{2}$. Here, $\varphi_{1}$ and $\varphi_{2}$ are the volume concentrations of PS and PA, respectively, the indices *′* and *″* refer to different coexisting phases, $r_{1}$ and $r_{2}$ are their degrees of polymerization.
- At the second stage, the critical value of the Flory–Huggins parameter *χ*_cr_ was calculated:
  (2) $$\chi_{\mathrm{cr}}=\frac{1}{2}{\left(\frac{1}{\sqrt{r_{1}}}+\frac{1}{\sqrt{r_{2}}}\right)}^{2}$$
- Then, the temperature dependences of the pair parameter were plotted in the coordinates *χ*–1/T (Figure 6), which were extrapolated to the values of *χ*_cr_. The point of intersection of these lines was taken for UCST.
- The critical concentration φ_cr_ was determined from the point of intersection of the Alekseev’s diameter and the isotherm corresponding to the UCST.
- The dome of the binodal curve and the spinodal curves separating the regions of metastable and labile solutions were calculated using the binodal and spinodal equations in the framework of the Flory–Huggins theory (shown in Figure 7).
- In addition, the temperature-concentration field of the diagrams contains limiting regions characterizing the region of thermal stability of homopolymers and their blends.

Figure 7 shows the generalized (complete) phase state diagrams, information about the UCST, critical compositions for the studying systems. It can be seen that UCST for mixtures of PS with PMA, PEA, PEHA is in the region of the onset of thermal degradation of oligomeric polystyrene (light gray zone) or acrylates (dark gray zone). It should be especially noted that the data on the thermal destruction of polyacrylates vary greatly. Thus, our studies on thermogravimetry have shown that a noticeable loss of polymer mass begins above 550 K, but at the same time, some structural changes can manifest themselves at 430 K. However, according to the data [12], PBA can undergo significant changes even when the temperature reaches 400 K.

It is fundamentally important that the ratio of the molecular weights of PA and PS, calculated from the value of the critical composition, are close to the experimentally found values, which indicates the effectiveness of the classical Flory–Huggins theory in describing (analyzing) the phase equilibrium of PS and PA blends.

The temperature dependences of the Flory–Huggins parameter (Figure 6) are linear in the coordinates *χ*−1/T. Mathematical expressions for such dependencies are determined by the method of least squares and are polynomials of the first degree. We have expanded the parameter *χ* into the entropy (first term of the polynomial) and enthalpy (second term of the polynomial) components (*χ*_S_ and *χ*_H_, respectively). The values of *χ*_H_ are characterized by the tangent of the slope of the straight lines *χ*−1/T and are close to each other for systems with one PS (straight lines 1–3 for PS-1 and straight lines 4–6 for PS-2) and they differ slightly when passing from PS-1 to PS-2. At the same time, *χ*_S_ for all systems is significantly different and decreases in the series PMA → PEA → PBA, which indicates a significant entropy contribution to the nature of mixing of the components.

**Figure 8:**
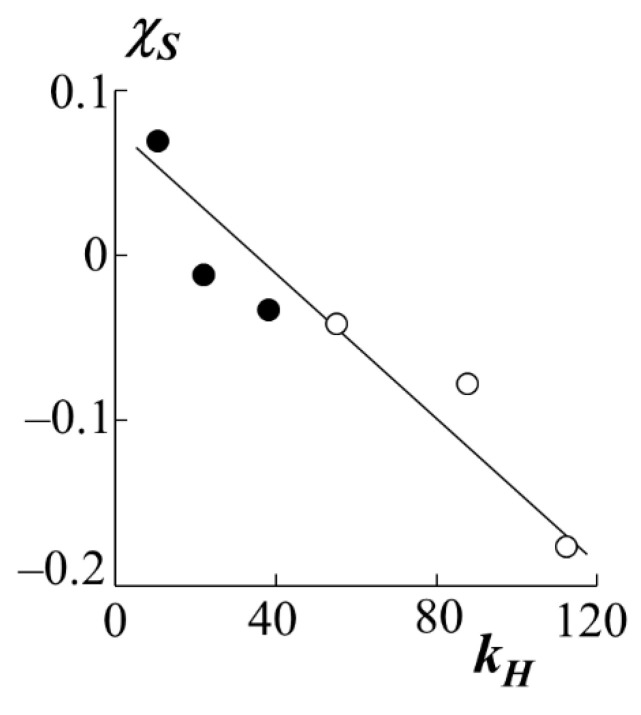
Ratio of entropic *χ*_S_ and enthalpy *k*_H_ components of the Flory–Huggins parameter for systems with PS-1 (white dots) and PS-2 (black dots).

If the enthalpy component of the interaction parameter is presented in the form χH=kHT, then it is possible to construct a dependence in the coordinates $\chi_{S}-k_{H}$ (Figure 8). Earlier, our colleagues tested the analysis technique [31], according to which most polymer-polymer systems are characterized by a linear dependence in these coordinates, which indicates the relationship between *χ*_S_ and *χ*_H_. Figure 8 shows that PS–PA systems behave in a similar way-a decrease in the entropy contribution leads to an increase in the enthalpy one and vice versa.

### 3.3. Diffusion

**Figure 9:**
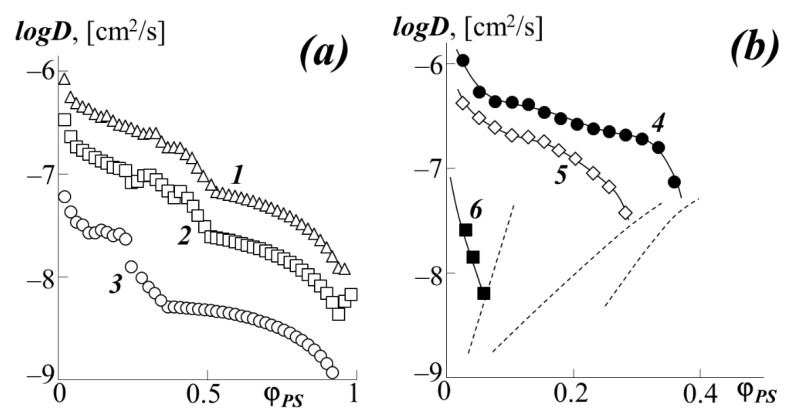
Concentration dependence of the diffusion coefficient at different temperatures (**a**) and for a number of acrylates at 433 K (**b**): 1–PBA–PS-1 at 433 K, 2–PBA–PS-1 at 413 K, 3–PBA–PS-1 at 373 K, 4–PEHA–PS-1, 5–PEA–PS-1, 6–PMA–PS-1. The dashed lines indicate the binodal curves of the corresponding systems.

Figure 9 shows the concentration dependences of the interdiffusion coefficients *D_V_* of the studied systems, calculated by the Matano–Boltzmann method [28,32]. Common to these dependences in the region of true solutions of phase state diagrams is their decrease as one approaches the boundaries of the two-phase region. This behavior of *D_V_* is associated with the presence of a labile region inside the amorphous delamination diagram (Figure 7) at the boundary of which the thermodynamic factor in Equation (3) vanishes.

(3) $$D_{V}=D^{*}\frac{\partial lna_{1}}{\partial ln\varphi_{1}}$$

Earlier, a similar effect was observed in the polyacrylates–solvents and polymethacrylates–solvents systems [33].

For the PBA–PS-1 system, the concentration dependence log*D_v_*–φ_1_ has a more complex form due to the competition between the effect of plasticization of polystyrene with polybutyl acrylate, which manifests itself in the concentration dependence of the partial diffusion coefficient D* and the concentration dependence of the thermodynamic factor. For example, at 433 K, when passing from the PS matrix (φ_PS_ = 1) to the PBA matrix (φ_PS_ = 0), the interdiffusion coefficient decreases by almost two decimal orders from −6.0 to −8.0 [cm^2^/s] (curve 1).

In the middle region of the compositions of the PBA–PS system, a slight extreme deviation of the curves from a monotonic decrease can be observed. From our point of view, this is due to the approach of the system to the "hidden" dome of the binodal curve, at the boundary of which, more precisely at the critical point, the thermodynamic factor in equation (3) vanishes.

An analysis of the concentration dependences of the interdiffusion coefficients showed that, when going from PS-1 to PS-2, its translational mobility in the PBA matrix significantly decreases (the numerical value of *logD* at φ_PS_ = 0.1 decreases from −6.4 to −7.1). In this case, the concentration dependence of the diffusion coefficient for the PBA–PS-2 system has a discontinuous character when approaching the region of phase decomposition of the system. The dependences for the diffusion of PS-1 into the matrix of various acrylates look similar (Figure 9b). Upon transition in the homologous series PBA → PEA → PMA (curves 1, 5, and 6, respectively), one can observe a decrease in the numerical values of *logD* by two orders of magnitude. There is also noticeable inflection and discontinuity of the curves at concentrations near the binodal curves (marked with a dashed line). It is interesting to note that up to φ_PS_ = 0.3, the PEHA–PS-1 system (curve 4) shows the same *logD* values and the same character of the curve as the PBA–PS-1 system (curve 1). At φ_PS_ > 0.3, instead of a monotonic decrease in the diffusion coefficient, as in the case of PBA, we see its sharp drop for PEHA in front of the binodal curve.

Analysis of the diffusion of polyacrylates into the PS matrix (right side of the phase diagram) is difficult due to the low solubility of the components in this region. Even from Figure 9a, it can be seen that the numerical value for the diffusion of PBA in PS is one to two orders of magnitude less than for PS diffusion into the PBA matrix and *logD* = −8 at 433 K *logD* = −9 at 373 K. When going from PBA to PMA, as well as from PS-1 to PS-2, the difference in diffusion coefficients in different matrices is leveled.

Information on the partial diffusion coefficients in extremely dilute solutions is of fundamental importance for the analysis of the mechanism of translational mobility of macromolecules in different media: at φ_PS_ → 0 the translation diffusion coefficients characterize the motion of PS macromolecules in the PA matrix, at φ_PS_ → 1 the motion of polyacrylate macromolecules in the PS matrix.

**Figure 10:**
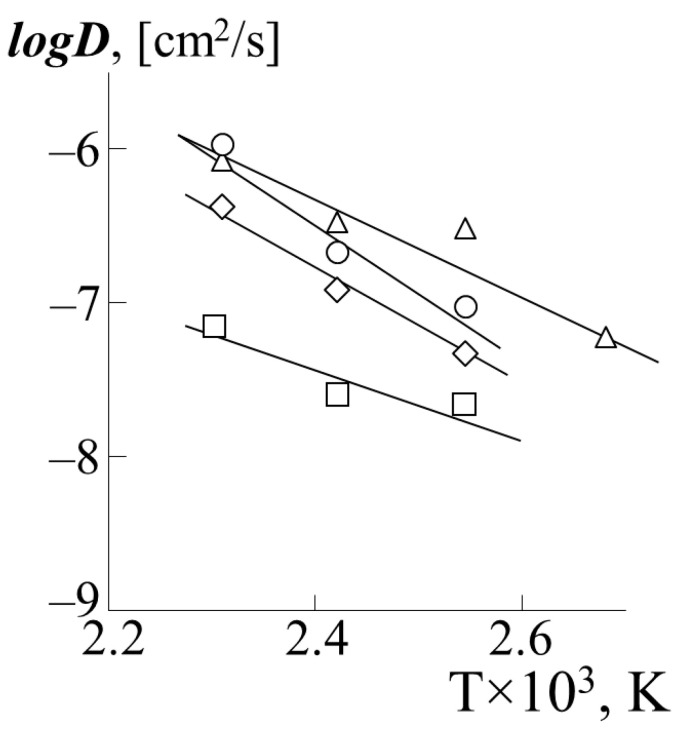
Temperature dependence of the partial diffusion coefficients of PS-1 into the matrix of PBA (triangle), PEA (rhombus), PMA (square) or PEHA (circle).

Figure 10 shows the temperature dependences of the partial diffusion coefficients of PS-1 into various PA in the coordinates of the Arrhenius equation (PS-2 behaves in a similar way). The calculated values of the diffusion activation energy are presented in Table 2. For comparison, the numerical value of the activation energy for self-diffusion of polystyrene with a molecular weight 7.5 × 10^4^ according to [23] is 75 kJ/mol.

**Figure 11:**
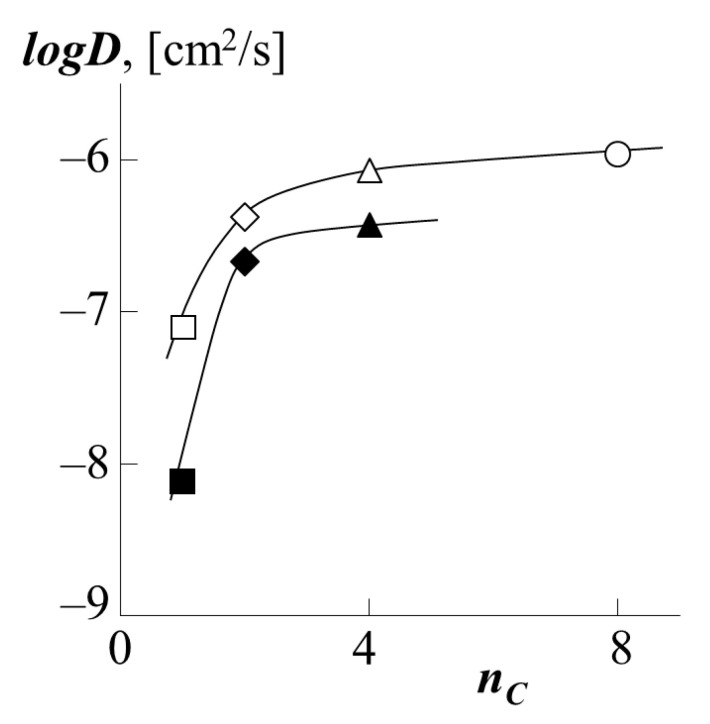
Dependence of the partial diffusion coefficients of PS-1 (white dots) or PS-2 (black dots) into the matrix of PBA (triangle), PEA (rhombus), PMA (square) or PEHA (circle) on the number of carbon atoms in the side chains polyacrylate.

To carry out a quantitative comparison of the effect of the type of polyacrylate on the translational mobility of PS macromolecules in it, by analogy with [34,35], we used such a parameter as the number of carbons *n_C_* in the side chain (functional group). The value of this parameter changes in the order PMA → PEA → PBA → PEHA as 1 → 2 → 4 → 8. Figure 11 shows the corresponding dependence of the diffusion coefficients. It is clearly seen that with an increase in the side chain length by one fragment (PMA → PEA), there is a sharp increase in the diffusion coefficient both for PS-1 as a diffusant and for PS-2 one. In the case of the transition from PEA to PBA, and then from PBA to PEHA, the dynamics of the growth rate of PS migration into the polyacrylate matrix are not so high. We assume that this is due to the conformational features of the packing of polyacrylate chains with different side chain lengths. Following [35], we assume that the short methyl group of PMA does not prevent dense packing of macromolecular coils. Longer fragments of side radicals of ethyl and butyl groups form a looser macromolecular coil of polyacrylate, which in turn increases the vacancy volume of the matrix and facilitates the migration of PS macromolecules into it. The same fact is indirectly indicated by the calculated values of the density of polyacrylates (Table 1). It is especially worth noting that the dependence shown in Figure 11 has a similar form in the coordinates *logD*–(*T-T*g), where (*T-T*g) is the difference between the experiment temperature and the glass transition temperature of PA.

To understand the nature of the movement of the PS macromolecular coil in the polyacrylate matrix forming the "tube" wall, we performed calculations in the framework of the reptational model [36]: the translational movement of macromolecules is described by the reptation model, within which the partial self-diffusion coefficient can be represented by an equation of the form

(4) $$D=\frac{kT}{Z}\frac{N_{e}}{N_{i}^{2}}$$

where *D* is self-diffusion coefficient, *Z* is the coefficient of friction of the chain per one link, which characterizes its movement in the "tube" of entanglements, *N_e_* is the number of links in the chain between the entanglements (taken equal to 100).

In our case, the self-diffusion coefficient of PS in polyacrylate and polyacrylate in PS depends on the local composition of the medium:

(5) $$D_{\Pi C}=\frac{kT}{Z_{\Pi C}}\frac{N_{e}}{N_{\Pi C}^{2}}$$

(6) $$D_{\Pi A}=\frac{kT}{Z_{\Pi A}}\frac{N_{e}}{N_{\Pi A}^{2}}$$

**Figure 12:**
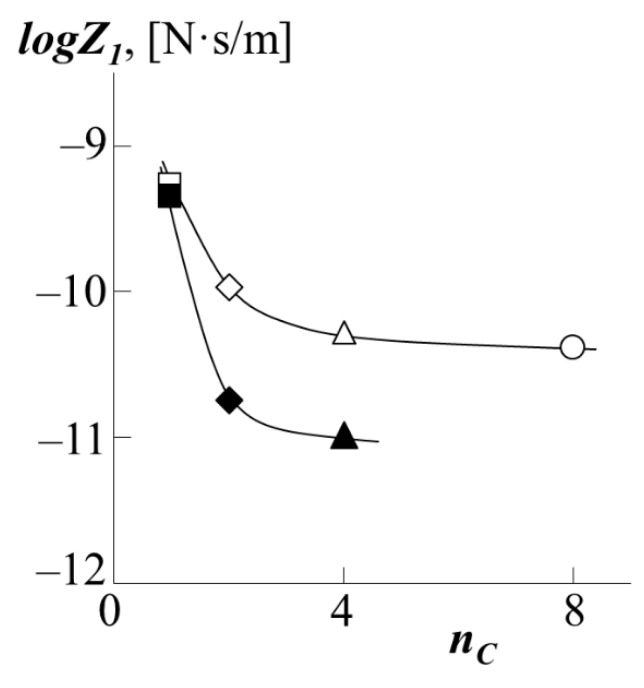
Dependence of the friction coefficients during the movement of PS-1 (white dots) or PS-2 (black dots) into the matrix of PBA (triangle), PEA (rhombus), PMA (square) or PEHA (circle) on the number of carbon atoms in the side chains of polyacrylate *n_C_* at 433 K.

Taking the boundary values of the interdiffusion coefficient as the self-diffusion coefficient, we were able to obtain approximate values of the chain friction coefficients for the case of the translational motion of the PS into polyacrylates (Figure 12). It can be seen that the friction coefficients of the PS chains, within the measurement error, monotonically decrease when passing from PMA matrix to PEA matrix forming the walls of the "tube". However, when going from PEA to PBA, the dependence reaches a plateau, showing that the diffusant is insensitive to structural changes within the diffusion medium. In our opinion, this confirms our assumption about the formation of a dense macromolecular coil for PMA and looser ones for other polyacrylates. In this case, during the diffusion of PS into PMA, the walls of the "tube" are formed between dense coils, creating a rougher relief of the surface of the "tube", the number of entanglements of the PS macromolecule is large, and hence the coefficient of friction is greater. On the contrary, in the case of the formation of a wide "tube" of loose macromolecular coils, the motion of PS is not hindered by additional entanglements, which leads to a decrease in the coefficient of friction. At the same time, the subsequent increase in the length of the side chain does not affect the reptational movement so strongly (plateau on the curve). The obtained results are in good agreement with the numerical values for the coefficient of friction when PS moves in PBA, which we published earlier [22]. However, it is interesting to note that during the diffusion of PS into a copolymer of styrene and butyl acrylate, the effect of the composition of the copolymer on the friction coefficient is directly opposite: the more styrene in the copolymer, the higher the friction coefficient.

**Figure 13:**
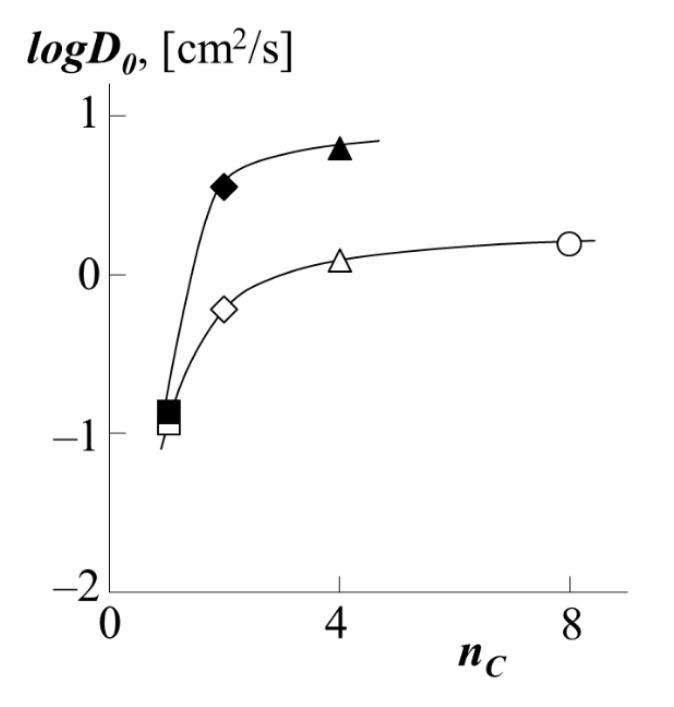
Dependence of the reptational constant *D*_0_ during the movement of PS-1 (white dots) or PS-2 (black dots) into the matrix of PBA (triangle), PEA (rhombus), PMA (square) or PEHA (circle) on the number of carbon atoms in side chains of *n_C_* polyacrylate at 433 K.

The conclusion about the contribution to the diffusion coefficient is also made possible by the information we obtained about the so-called reptational constant D0=kTZ, which has the meaning of the self-diffusion coefficient for a broken link system in which the monomer links are not linked in a chain (Figure 13).

## 4. Conclusions

We have shown that the solubility upon mixing PS with polyacrylates improves with an increase in the length of the side fragment of the polyacrylate functional group, but worsens with an increase in M_PS_. However, if the PBA–PS-1 system is completely mutually soluble, then the other studied systems are characterized by phase state diagrams with UCST. In this case, the solubility of oligomeric PS in polyacrylates is significantly higher than that of polyacrylates in PS. These data correlate well with the data on phase equilibrium upon mixing PS with styrene and butyl acrylate copolymers that we obtained earlier.

The binodal curves were used to determine and analyze the pair interaction parameters and their temperature dependences in the framework of the Flory–Huggins theory. Decomposition of the interaction parameter into enthalpy and entropy components showed that the first does not change when passing from one homologue to another in the polyacrylates series, in contrast to the second one. However, we can definitely speak of a linear relationship between *χ*_S_ and *χ*_H_, which is typical for most polymer-polymer systems.

We have calculated the diffusion coefficients during the movement of polystyrene into polyacrylates and polyacrylates into polystyrene. An analysis of their concentration dependence showed that with an increase in the content of PS in the blend, the numerical values of the interaction diffusion coefficients significantly decrease due to the large difference in the self-diffusion coefficients of polyacrylates and PS. In this case, when approaching along the concentration axis to the region of phase decomposition, the concentration dependence of the interdiffusion coefficients has a discontinuous character. The transition from PS-1 to PS-2 significantly reduces the rate of interdiffusion of the components (at φ_PS_ = 0.1 logD changes from −6.4 to −7.2 at 433 K when mixed with PBA). The change in polyacrylate in the homologous series PMA → PEA → PBA (at φ_PS_ = 0.1 logD changes from −8.5 → −6.7 → −6.4 at 433 K when mixed with PS-1) has an opposite effect. Analysis of the dependences of the diffusion coefficients of PS into the polyacrylate matrix on the number of carbon atoms in the side chain of the polyacrylate showed that they are characterized by a curve with saturation. We assume that this is due to the formation of a dense macromolecular coil in PMA and looser coils in its other homologues. Calculations of the friction coefficients in the framework of the theory of reptational movement showed that the PS macromolecule, when moving in a denser and rougher "tube" of the PMA matrix, has more engagement with the walls of such a “tube” (the friction coefficient is higher). However, the transition from PEA to PBA does not have the same strong effect as the transition from PMA to PEA. The temperature dependences of the interdiffusion coefficients were used to estimate the activation energies of mixing, the numerical values of which vary from 12.9 to 15.7 kJ/mol upon transition in the series PMA → PEA → PBA during diffusion of PS-1 and from 20.1 to 34.7 kJ/mol upon transition in the series PMA → PEA → PBA with PS-2 diffusion.

## Figures and Tables

**Table 1:** The characteristics of the studied objects.

Polymer	Brand	M_w_	M_w_/M_n_	T_g_ *, K	n_C_	M_fg_	ρ **, g/cm^3^
PS-1	PSS	1220	1.07	343	-	104	1.04
PS-2	Aldrich	4120	1.01	353	-	104	1.04
PMA	NII Polymerov Dzerzhinsk, Russia	32,921	4.47	282	1	86	1.22
PEA	NII Polymerov Dzerzhinsk, Russia	21,437	3.00	250	2	100	1.12
PBA	NII Polymerov Dzerzhinsk, Russia	26,195	3.33	218	4	128	1.04
PEHA	NII Polymerov Dzerzhinsk, Russia	19,824	2.86	188	8	140	0.74

* according to differential scanning calorimetry data (Netzsch DSC 204 F1 Phoenix, Germany). ** densities were calculated from the experimental values of the refractive index at 298 K and the group contributions to the Lorentz-Lorenz molar refraction [25].

**Table 2:** Activation energy of diffusion of PS-1 and PS-2 macromolecules into polyacrylates.

System	E_a_, kJ/mol	System	E_a_, kJ/mol
PBA–PS-1	15.4	PBA–PS-2	34.7
PEA–PS-1	14.1	PEA–PS-2	26.4
PMA–PS-1	12.9	PMA–PS-2	20.1
PEHA–PS-1	20.1		

## Data Availability

The data presented in this study are available on request from the corresponding author.
